# Analysis of Delta(9) fatty acid desaturase gene family and their role in oleic acid accumulation in *Carya cathayensis* kernel

**DOI:** 10.3389/fpls.2023.1193063

**Published:** 2023-09-12

**Authors:** Xiaolin Si, Shiheng Lyu, Quaid Hussain, Hongyu Ye, Chunying Huang, Yan Li, Jianqin Huang, Jianjun Chen, Ketao Wang

**Affiliations:** ^1^ State Key Laboratory of Subtropical Silviculture, Zhejiang A&F University, Lin’an, Zhejiang, China; ^2^ College of Life Sciences and Oceanography, Shenzhen University, Shenzhen, China; ^3^ Mid-Florida Research and Education Center, Environmental Horticulture Department, Institute of Food and Agricultural Sciences, University of Florida, Apopka, FL, United States

**Keywords:** hickory, stearoyl-ACP-desaturase, oleic acid, fatty acid composition, molecular docking

## Abstract

*Carya cathayensis*, commonly referred to as Chinese hickory, produces nuts that contain high-quality edible oils, particularly oleic acid (18:1). It is known that stearoyl-ACP desaturase (SAD) is the first key step converting stearic acid (C18:0, SA) to oleic acid (C18:1, OA) in the aminolevulinic acid (ALA) biosynthetic pathway and play an important role in OA accumulation. Thus far, there is little information about *SAD* gene family in *C. cathayensis* and the role of individual members in OA accumulation. This study searched the Chinese Hickory Genome Database and identified five members of *SAD* genes, designated as *CcSADs*, at the whole genome level through the comparison with the homologous genes from *Arabidopsis*. RNA-Seq analysis showed that *CcSSI2-1, CcSSI2-2*, and *CcSAD6* were highly expressed in kernels. The expression pattern of *CcSADs* was significantly correlated with fatty acid accumulation during the kernel development. In addition, five full-length cDNAs encoding *SADs* were isolated from the developing kernel of *C. cathayensis*. CcSADs-green fluorescent protein (GFP) fusion construct was infiltrated into tobacco epidermal cells, and results indicated their chloroplast localization. The catalytic function of these *CcSADs* was further analyzed by heterologous expression in *Saccharomyces cerevisiae*, *Nicotiana benthamiana*, and walnut. Functional analysis demonstrated that all *CcSADs* had fatty acid desaturase activity to catalyze oleic acid biosynthesis. Some members of *CcSADs* also have strong substrate specificity for 16:0-ACP to synthesize palmitoleic acid (C16:1, PA). Our study documented *SAD* gene family in *C. cathayensis* and the role of *CcSSI2-1, CcSSI2-2*, and *CcSAD6* in OA accumulation, which could be important for future improvement of OA content in this species via genetic manipulation.

## Introduction

*Carya cathayensis* Sarg., commonly known as Chinese hickory, is an economically important species in the family Juglandaceae. It is a deciduous, nut tree and widely cultivated in the mountainous areas of Zhejiang and Anhui provinces, China (Grauke et al., 2016; Huang et al., 2019). The kernel of Chinese hickory is rich in fat and proteins and contributes substantially to its nutritional value. Processing nuts at a high temperature or roasting results in the release of a unique aroma and flavor with a taste like mild pecans, which attracts numerous consumers. The food industry also uses roasted nuts as snacks and ingredients of various candies and cakes (Valdés García et al., 2021). Oil extracted from nuts is used in cooking. With the awareness of its value, there is increasing research on this species, including grafting propagation (Xu et al., 2017), flower development (Fan et al., 2020), genomic and transcriptomic analysis of its fruit development (Huang et al., 2019; Huang et al., 2020), nut quality (Chen et al., 2021; Chen et al., 2022), and tolerance to abiotic and biotic stresses (Sharma et al., 2020; Wu et al., 2020; Ma et al., 2023).

A distinct characteristic of Chinese hickory nut is its high oil content. Hickory accumulates more than 70% oil in the mature embryo, which is higher than the other major oilseed crops, such as rapeseeds (40–50%), peanut (35–40%), or oil palm (30– 50%) (Huang et al., 2015; Huang et al., 2022a). To gain information on the high oil accumulation, global transcriptomic and lipidomic analyses were conducted to pursue a better understanding of embryogenesis, seed filling, and maturation processes as well as seed quality in the hickory nut, and results showed that transcripts of malonyl-CoA (Kachroo and Kachroo, 2009), a key intermediary metabolite in fatty acid (FA) synthesis contributes significantly to triacylglycerol (TAG) assembly and oil body formation (Huang et al., 2016). Subsequent combined proteomic and transcriptomic analyses suggested essential metabolic genes or enzymes are important for the biosynthesis and accumulation of TAGs from sugars in multiple pathways (Huang et al., 2022a).

A unique property of Chinese hickory oil is its high percentage of unsaturated fatty acids. The saturated fatty acids including lauric, myristic, palmitic acid (16:0), stearic acid (18:0), and arachidic acid (20:0); and the unsaturated fatty acids, such as oleic acid (OA,18:1^Δ9^), linoleic acid (18:2^Δ9,12^), linolenic acid (18:3^Δ9,12,15^), 11-eicosenoic acid (20:1^Δ11^), and erucic acid (22:1^Δ13^) together account for almost the entire fatty acid content of higher plants. According to the position of the double bonds, the unsaturated fatty acids include ω-3, ω-6, ω-7 or ω-9 types (Dyer et al., 2008). The oil-rich kernel of *C. cathayensis* is composed of 90% monounsaturated and polyunsaturated fatty acids (FAs), especially ω-3 and ω-6 polyunsaturated fatty acids with high nutritional value and health benefits FAs (Huang et al., 2016). On the other hand, consumption of saturated fatty acids increases overall cholesterol levels, specifically low-density lipoprotein (LDL) or “bad” cholesterol, leading to an increased risk of cardiovascular disease (Baum et al., 2012). Thus, a rational fatty acid composition is often found in higher-quality nuts for daily human oil supplementation.

Moreover, Chinese hickory oil has a high level of oleic acid (OA) (∼80%) and low level of linoleic acid (18:2), which gives what is regarded as the most suitable ratio of OA/linoleic acid for human health and is comparable to that of walnut. Due to the presence of polyunsaturated fatty acids, oil in walnut is chemically unstable and susceptible to oxidative deterioration, especially when exposed to oxygen, light, high moisture, and temperature. Oxidative degradation of linoleic acid results in a loss of nutritional quality and the development of undesirable flavors, affecting the oil’s shelf stability and sensory properties (Waraho et al., 2011). On the contrary, OA has remarkable oxidative stability and physicochemical properties. The lipidomics profile of *C. cathayensis* nuts indicated that TAGs, diacylglycerols, phosphatidylethanolamines, and phosphatidylcholines had high relative content with an abundance of unsaturated fatty acids, specifically oleic acid, linoleic acid, and linolenic acid, localized mainly at sn-2 lipid position (Huang et al., 2022b). Notably, lipid extracts from hickory nuts could promote the synaptic growth of SH-SY5Y cells, offering possible evidence that an appropriate ratio of unsaturated fatty acids can improve memory ability and delay neuronal degeneration in humans (Gao et al., 2020). Fatty acids are an essential source of energy in nuts and important precursors for the formation of specific flavors. Oils enriched in OA have significant industrial value due to their stable chemical properties and high combustion quality, allowing their direct use as a renewable feedstock for biolubricants, biodiesel, and other oleochemical-based polymers (Rosenboom et al., 2022). Thus, it could be highly desirable to reduce the polyunsaturated fatty acid content and increase the OA content (18:1^Δ9^). However, the physiological and molecular mechanisms underlying oleic acid accumulation in *C. cathayensis* is still unclear.

As a key enzyme in plant lipid metabolism, fatty acid desaturase (FADs) can introduce double bonds to specific positions of FAs and convert the saturated FAs into unsaturated ones in the plastid and the endoplasmic reticulum (ER) (Ohlrogge and Browse, 1995). The stearoyl-ACP desaturase (FAB2/SAD) is known as the only soluble FADs in the plastid matrix catalyzing the first desaturation step through the conversion of stearic acid (C18: 0) to oleic acid (C18: 1) by adding a cis-double bond between C9 and C10 of the carbon chain (Kazaz et al., 2020; Kazaz et al., 2022). Thus, SADs play an important role in determining seed oil content and oil composition, significantly affecting the ratio of saturated to unsaturated fatty acids. Because of the functional importance of FA saturation in plant development and industrial applications, homologous SAD genes have been cloned and characterized from many plant species (Knutzon et al., 1992; Zaborowska et al., 2002; Liu et al., 2009; Shilman et al., 2010; Wang et al., 2012; Pan et al., 2013; Liu et al., 2019a; Cappetta et al., 2022). Silencing expression of the *GhSAD* gene can significantly reduce oleic acid content in cottonseed oil from ~13% to ~4%, and increase steric acid content from ~2% to ~40% (Liu et al., 2002). Similarly, in six soybean mutants, *SACPD-C* mutation led to elevated stearic acid content in soybean seeds that was 1.5 to 3-fold of the wild type (Carrero-Colon et al., 2014; Lakhssassi et al., 2017; Lakhssassi etal., 2020; Ruddle et al., 2014). Overexpressing *S-ACP-DES1* gene in the *ssi2* Arabidopsis mutant restored its capacity of catalyzing the production of oleic acid (18:1^Δ9^) (Kachroo et al., 2007). Overexpression of *Lupinus luteus* L. *SAD* gene substantially increased the oleic acid content in tobacco leaves (Zaborowska et al., 2002). However, data on the expression diversity of these genes and their influence on oil composition are still insufficient, which hampers the development of the marker-assisted selection. Moreover, these studies largely focused on one or two *SAD* genes without functional characterization, and results often directly correlated gene expression levels with biological activity, which provided limited information on OA accumulation in seeds.

Recently, the availability of genomic data has greatly facilitated the identification of genes of interest. As the Chinese Hickory Genome Database is available, this study was intended to identify *SAD* genes at the whole genome level in *C. cathayensis*, comprehensively analyze the SAD gene family, and identified *SADs* were further analyzed by heterologous expression in *Saccharomyces cerevisiae*, *Nicotiana benthamiana*, and walnut. Further research on the specific biological functions and substrate selectivity of these differentially expressed *SAD* family members will help comprehensively analyze the regulatory mechanism and improve plant oil quality. Our data showed that the identified *SADs* exhibited fatty acid desaturase activity by catalyzing oleic acid biosynthesis.

## Materials and methods

### Identification of *SAD* genes and their protein prediction

To identify *SAD* genes in *C. cathayensis*, the coding sequence and amino acid sequence of *SADs* from different plants (*Arabidopsis thaliana*, *Oryza sativa* L., and *Populus* L.) were used as queries to search against the Chinese Hickory Genome Database. The whole genomic sequences of hickory (*C. cathyensis*) were obtained from the Portal of Juglandaceae (Guo et al., 2020; Yang et al., 2016). The SAD protein sequence data of *A. thaliana*, *Populus* and *O. sativa* were downloaded from the Arabidopsis Information Resource (TAIR) database (http://www.arabidopsis.org), the China Rice Data Center (http://www.ricedata.cn/gene/index.htm) and the previous studies, respectively. Using Arabidopsis, rice, and poplar *SAD* gene family members as queries to search homologous protein sequences in the hickory protein database by using the Blast Zone program (TBtools-blast software, E-value <1e-10) (Chen et al., 2020), respectively.

All candidate SACPD (stearoyl-acyl carrier protein desaturase) proteins were analyzed using HMMER software to identify the FA_desaturase_2 domain with the Pfam accession PF03405 (Finn et al., 2015; Mistry et al., 2020). They were further validated using the NCBI Conserved Domain Database (http://www.ncbi.nlm.nih.gov/Structure/cdd/wrpsb.cgi), SMART (http://smart.embl-heidelberg.de/), and InterProScan programs (http://www.ebi.ac.uk/Tools/InterProScan/) (Jones et al., 2014).

### Multiple sequence alignment and analysis of phylogenetic tree

Multiple sequence alignment and visualization analyses were performed based on the amino acid sequences of CcSADs and AtSADs. Then, the phylogenetic tree of all SAD proteins was constructed by MEGA7 using the neighbor-joining (NJ) method with bootstrap values of 1,000 replicates (Kumar et al., 2016). All isolated CcSAD (*C. cathayensis* SAD) proteins were classified into different subfamilies based on the SADs from different species. The nomenclature of CcSADs are based on the similarity of amino acid sequences to homologous genes in *Arabidopsis* including SAD and SSI2 (SUPPRESSOR OF SA-INSENSITIVITY2)

### Analyses of physiochemical properties and subcellular localization of CcSAD

The ExPASy: SIB Bioinformatics Resource Portal-Home (https://www.expasy.org/) was used to predict the theoretical isoelectric point (pI) and molecular weight (MW) of CcSAD proteins. The instability index (II), aliphatic index, and grand average of hydropathicity (GRAVY) were analyzed using the ProtParam tool of the ExPASy website (https://web.expasy.org/protparam/) (Gasteiger, 2005). The subcellular locations of CcSAD proteins were predicted using WoLF PSORT and ProtCompV.9.0 Server (http://www.soft-berry.com) (Horton et al., 2007). The presence of signal peptide and transmembrane domains of CcSAD proteins was predicted using SignalP 4.0 and TMHMM v.2.0 online software.

### Analyses of gene structure, domain and conserved motif compositions of CcSADs

The conserved motifs of CcSAD proteins were analyzed using MEME-Suite 5.4.1 online program (http://meme-suite.org/) (Bailey et al., 2015). The width of the conserved motifs was set to have 6 to 50 amino acids, and the maximum number of conserved sequences was set to be 10. Finally, the conserved motif, gene structure, and conserved domain of the *CcSAD* gene family were visualized using TB tools (Chen et al., 2020).

### Cis-element analysis of *CcSADs*

To identify the cis-elements of *CcSADs*, the upstream 2,000 bp sequences from the translation start site were collected from the Chinese Hickory Genome Database and submitted to the PlantCARE tool (http://bioinformatics.psb.ugent.be/webtools/plantcare/html/) for cis-element sequence search, The identified cis-regulatory elements were analyzed according to the method of Lescot et al. (2002).

### Gene and protein expression analysis

Previous transcriptomic analyses documented expression patterns of *CcSAD* genes during different stages of embryo development (Huang et al., 2022a) and also in stigma (Xing et al., 2022) and endocarp (Li et al., 2022b) when *C. cathayensis* plants were grown under normal conditions. Thus, raw RNA-Seq data for *C. cathayensis* embryo, stigma, and endocarp were downloaded from the NCBI created by our lab (Read Archive accession number: PRJNA687050, PRJNA810757). Poly N, adaptor sequences, and low-quality reads were removed and assembled by Trinity software to obtain clean data. The clean reads from each sample were mapped to reference genome of Chinese Hickory (Chinese Hickory Genome Database) using Hisat2. Expression levels of the genes were measured as fragments per kilobase of transcript per million mapped reads (FPKM). The expression data of SAD genes were log2-transformed to normalize the scale by Tbtools (Scale Method: Zero To One). Subsequently, correlation coefficients between variation in unsaturated fatty acids and SAD genes expression were computed using the R language. The software Mascot 2.3.02 (Matrix Science, UK) was employed for protein analysis, and data were downloaded from the NCBI (Read Archive accession number: PRJNA687050)

### Molecular ducking

The ligand spatial data (FAs: C16:/C17:0/C18:0) were obtained from the PubChem database (https://pubchem.ncbi.nlm.nih.gov/). The protein structure and amino acid sequences of CcSADs were predicted using an online tool AlphaFold Colab (https://colab.research.google.com/github/sokrypton/ColabFold/blob/main/AlphaFold2.ipynb). The protein structure was introduced into Autodock tools to remove the water molecules from the pdb add hydrogens. Using CB-DOCK2 (http://cadd.labshare.cn/cb-dock2/), protein file (pdb format) and ligand file (pdb format) were loaded into the specified area, and molecular docking was predicted (Salmaso and Moro, 2018; Liu et al., 2022).

### Gene cloning and plasmid construction

Total RNA was extracted from the endosperm of immature seeds using EasyPure® Plant RNA Kit (Transgen, Beijing, ER301-01) and was reverse-transcribed to cDNA using the EasyScript® First-Strand cDNA Synthesis SuperMix (Transgen, Beijing, AE301-02). Using TransStart® FastPfu Fly DNA Polymerase (Transgen, Beijing, AP231-21), PCR was performed using the cDNA as a template to generate *CcSAD* amplicons for subsequent sequencing analysis. An EasyPure® Quick Gel Extraction Kit (Transgen, Beijing) was used to purify the PCR products. All primer sequences used in this paper were in the **Supplementary File Table S1**.

The CcSADs without the stop codon were cloned into the BamHI site of the pCambia1300-35S-EGFP vector to generate a 35S-CcSAD-GFP construct so that these genes could fuse with the GFP protein driven by the 35S promoter when expressed in tobacco (*N. benthamiana*) leaves (Craig et al., 2008). Primers with homologous arms and related enzyme digestion sites were used to amplify the full-length CDS of *CcSAD*, which was then inserted into the pCambia1300-35S-EGFP vector with a pEASY®-Basic Seamless Cloning and Assembly Kit (Transgen, Beijing, CU201-02). In addition, the coding sequences of *CcSADs* were amplified by PCR to generate pDR195-CcSAD yeast expression vectors using the pEASY®-Basic Seamless Cloning and Assembly Kit (Transgen, Beijing) on a BamHI-treated pDR195 vector.

### Transient transformation to tobacco leaves and subcellular localization

The correctly sequenced 35S-CcSADs-GFP construct was transferred into *Agrobacterium tumefaciens* strain GV3101, which was transformed into tobacco leaves through infiltration. Two days after infiltration, the transient expression of GFP in tobacco leaves was observed using a Confocal Laser Scanning Microscopy (ZEISS LCM-800, Carl Zeiss, Oberkochen, Germany) with 470 nm excitation filter and 525 nm emission filter.

### Isolation and transformation of Arabidopsis protoplasts

Mesophyll protoplasts were isolated from *A. thaliana* leaves. Briefly, the cut leaf strips were mixed with an enzymatic digestion solution containing mannitol, CaCl2·2H2O, MES, macerozyme R10, and cellulase R10. The mixture was incubated in the dark at room temperature for at least 3 hours. After digestion, the protoplasts in the solution were examined under a microscope. After the incubation, protoplasts were harvested by filtration with 0.65-μm nylon filters and centrifugation at 100 g for 5 min. The supernatant was removed, and protoplasts were gently resuspended in pre-chilled W5 solution (2 mM MES, 154 mM NaCl, 125 mM CaCl2, and 5 mM KCl) on ice. The resulting pellet was resuspended in MMG solution (Mannitol, MgCl2·6H2O, and MES). The vitality of the protoplasts was observed using a microscope, and the concentration of protoplasts was adjusted to 2 ×10^5^ protoplasts/mL. Protoplasts were transiently transformed using 5 mg of 35S-CcSAD-GFP plasmid with chloroplast marker RUB1sp-mCherry by PEG solution (40% PEG6000, 100 mM glucose, 10 mM CaCl2 and 0.7 mM KH2PO4 at pH 5.8).

### Functional analysis of CcSADs in yeast

Using the PEG/lithium acetate method (Gietz and Schiestl, 2007), the recombinant plasmids of pDR195 containing PMA1::CcSADs and pDR195 control (an empty vector) were transformed into *S. cerevisiae* strain BY4389, respectively to express proteins in transgenic yeast. *S. cerevisiae* transformants were selected on SD/-Ura minimal medium, and the positive clones with pDR195 (the control vector) or pDR195/PMA1::CcSADs were grown in SD/-Ura liquid medium at 30°C with sharking. When the OD600 value reached 0.8, OA or LA was added synchronously as a substrate for yeast cells. After being cultured at 30°C for 72 h, the cells were centrifuged at 4000 ×g for 10 min and washed with sterile water three times. The thalli were freeze-dried at -80°C overnight, and 10 mg of freeze-dried yeast was weighed into a 1.5 mL centrifuge tube, 200 μL of a 1 mg/mL C17:0 hexadecanoic acid hexane solution and 5 mg of glass beads were added. The 1.5 mL centrifuge tube was shaken for five min and then sonicated for 10 min for total lipid extraction. Samples were then centrifuged at 12000r/min for 10 min, and the supernatant was collected and stored at -80°C for FA content analysis.

### Transient transformation to walnut

The 35S-CcSAD-GFP construct recombinant plasmids were transferred into an *Agrobacterium tumefaciens* strain (GV3101) using the freeze-thaw method. The strain was then cultured in 20 mL of Luria-Bertani (LB) medium at 28°C for 16 hours with shaking at 200 rpm. Walnuts were submerged in 10% sodium hypochlorite (NaClO) and placed in a vacuum pump for 15 minutes. Afterward, the walnuts were washed 2-3 times with sterile water. The fruits were then peeled, and the embryos were placed in WPM medium. Embryos cultured in WPM medium were transferred into the bacterial liquid of *A. tumefaciens* with an OD600 value of 0.8. After 15-20 minutes of co-cultivation, the embryos were transferred to paper towels to dry. Once dry, the embryos were transferred to MS medium containing 100 µM As for incubation.

### Fatty acid transmethylation and GC–FID analysis

Chinese hickory stems, roots, leaves, and fruits, somatic embryos of walnut, and tobacco leaves were harvested for GC–FID analysis, all these plants were grown in normal conditions. The collected samples were transmethylated in 1 mL methanol containing 2% H_2_SO_4_ (v/v) and 50 μg butylated hydroxytoluene, and 300 μg of heptadecanoic acid (C17:0) was added to each sample as an internal standard. Transmethylation was maintained in a heated water bath (85°C) for 1.5 h and rapidly cooled in an ice box. Then 2 mL of 9% NaCl solution (m/v) and 2 mL of n-hexane were added for extracting FA methyl esters. Finally, the supernatant was used for analyzing FA components using DB-FASTFAME (Agilent). Nitrogen was used as the carrier gas in constant pressure mode at 28 psi. 1 µL of the sample was injected into the column and repeated three times. The inlet temperature was set at 250°C, the detector temperature was 260°C, and the GC oven was initially set at 80°C (hold for 0.5 min), ramped up to 175°C at 65°C/min, to 185°C at 10°C/min (hold for 0.5 min), and finally to 230°C at 7°C/min. Fatty acid fractions were estimated from the retention times of the fatty acid standards, and data were collected by peak area normalization using the C17:0 internal standard.

### Statistical analysis

All the data were presented as means ± SE (n = 3) and statistically analyzed using SPSS 22.0 (SPSS Incorporated, USA). Mean differences between groups were analyzed using Student’s *t*-test.

## Results

### Identification of *CcSAD* genes

Five SAD proteins were identified in Chinese hickory and named as CcSADs based on their similarity to *Arabidopsis* SAD proteins. They were further characterized based on their molecular weight, isoelectric point, amino acid, instability index, aliphatic index, and grand average of hydropathicity, respectively (**Table 1**). The deduced CcSADs lengths varied from 289 to 396 amino acids. The molecular weight of CcSAD proteins ranged from 32.48 kDa to 45.22 kDa. CcSSI2-2 has a higher molecular weight of 45.22 kDa, followed by CcSSI2-1 with 45.57 kDa. CcSAD6 has the lowest molecular weight of 32.48 kDa among the CcSAD proteins.

**Table 1:** Physicochemical properties of SAD proteins derived from *Carya cathayensis*.

Sequence ID	Number of Amino Acid	Molecular Weight	Theoretical pI	Instability Index	Aliphatic Index	Grand Average of Hydropathicity	Subcellular Localization Prediction	Number of predicted TMHs	Signal Peptide
CcSSI2-1	396	45102.63	6.24	35.91	78.61	-0.445	Chloroplast	0	No
CcSSI2-2	396	45225.73	6.29	38.68	80.1	-0.449	Chloroplast	0	No
CcSAD2	389	44521.92	8.72	45.24	79.02	-0.459	Chloroplast	0	No
CcSAD4	389	44436.73	7.23	44.36	78.02	-0.454	Chloroplast	0	No
CcSAD6	289	32483.23	5.38	23.86	86.09	-0.22	Cytoplasmic	0	No

**Figure 1:**
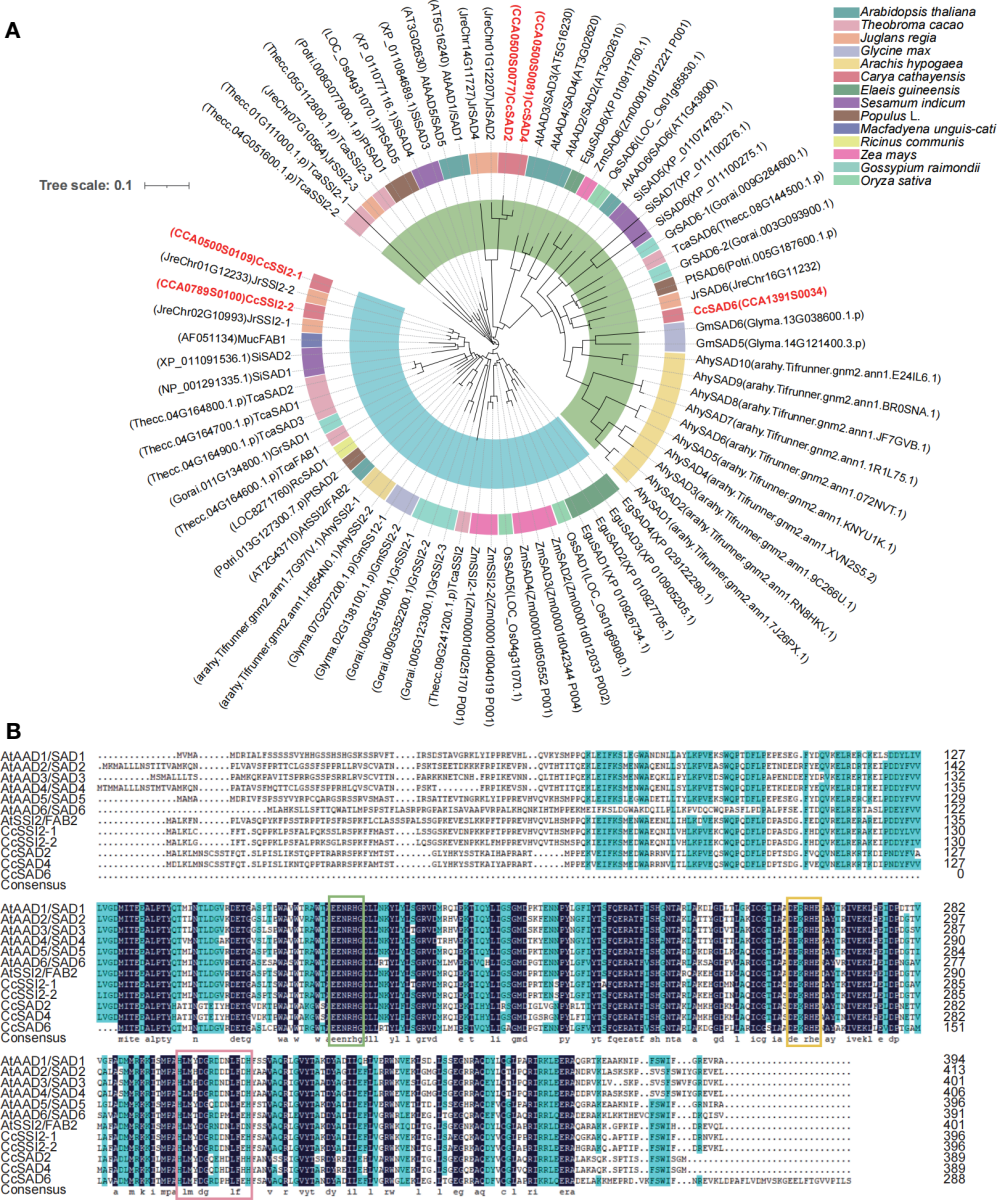
Phylogenetic tree and multiple sequence alignment of SAD proteins from *Carya cathayensis* and other plant species. **(A)** Phylogenetic tree analysis of SADs from *C. cathayensis* and other higher plants including *Arabidopsis thaliana, Theobroma cacao, Juglans regia, Glycine max, Arachis hypogaea, Carya cathayensis, Elaeis guineensis, Sesamum indicum, Populus* spp.*, Macfadyena unguis, Ricinus communis, Zea mays, Oryza sativa*, and *Gossypium raimondii*. The tree was constructed using the neighbor-joining method with 1,000 bootstrap replicates. The numbers of individual clades correspond to bootstrap support values. The inside blue and green shadings represent two different groups. **(B)** Amino acid sequence alignment of CcSAD from *C*. *cathayensis* and *A*. *thaliana.* The features of the sequence include histidine boxes, EENRHG, and DEKRHE boxes highlighted by lines above the alignment.

To characterize the phylogenetic relationship among SADs from different plants and Chinese hickory, a neighbor-joining (NJ) tree was constructed using the MEGA software based on the alignment of five CcSADs in *C. cathayensis*, seven AtSADs in *Arabidopsis thaliana*, five PtSADs in *Populus*, and SAD proteins from other plants (**Figure 1A**). Topology of the phylogenetic tree indicated that the SAD gene family could be divided into two subgroups indicated by blue and green shading, respectively. We also performed a progressive alignment of SAD gene sequences from *C. cathayensis* and *Arabidopsis*, resulting in a multiple alignment (**Figure 1B**). The putative two conserved peptides (DETGASP and DYADILE) and the E/DEXXH motifs are denoted with boxes.

### Gene structure and conserved domains analysis

**Figure 2:**
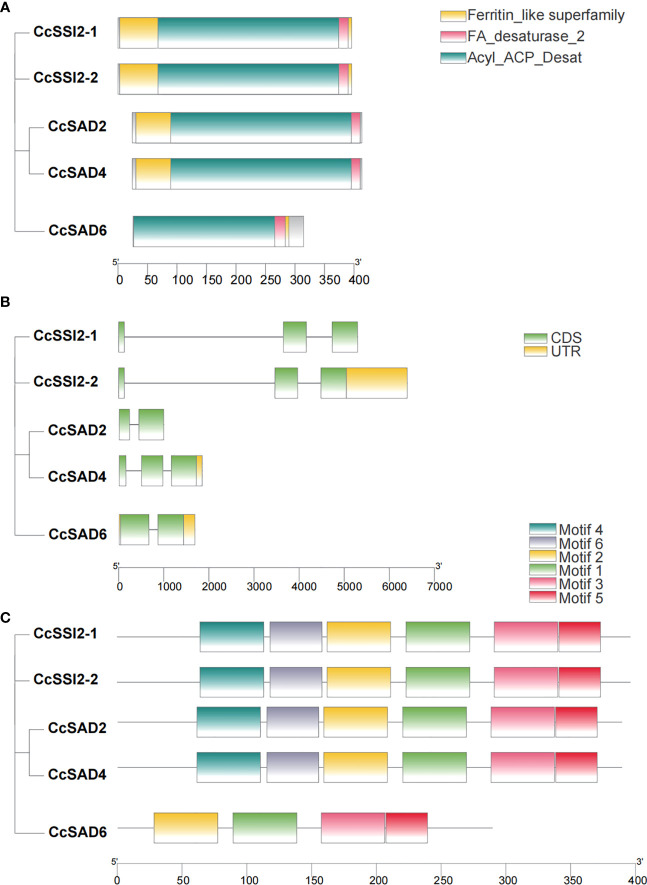
Characterization of SAD proteins from *Carya cathayensis*. **(A)** Conserved domain of SAD genes in *C*. *cathayensis.* **(B)** Gene structure of SAD genes in *C*. *cathayensis* in which green boxes represent exon, and black lines represent introns. **(C)** Conserved motif of SAD genes in *C*. *cathayensis* where different colors indicate different motifs.

To clarify the evolutionary relationships, gene structure, conserved motifs, and conserved domains of five *CcSAD* genes were analyzed. Phylogenetic analysis indicated that CcSADs proteins had two subfamilies, which was similar to the topological structure of a phylogenetic tree in **Figure 1A**. Except for CcSAD6, all CcSADs had FA desaturase_2, Acyl_ACP_Desat conserved domains and Ferritin like superfamily domain (**Figure 2A**). By comparing the CDS of *CcSADs* with the genome sequence of the corresponding genes, the UTR, CDS, and intron distribution of CcSADs were analyzed in depth (**Figure 2B**). There was one intron in the two members *CcSADs* (*CcSAD2* and *CcSAD6*). Members of the same subfamily showed similar exon/intron distribution patterns, such as CcSSI2-1 and CcSSI2-2 having two introns with a long first intron (**Figure 2B**). There were two conserved histidine rich regions which is a typical *SAD* characteristic, namely EENRHG and DEKRHE where aspartate (D) and histidine (H) provide necessary binding sites for ferric ions in the catalytic active center of *CcSAD*, ensuring that dehydrogenase has certain catalytic activity. Remarkably, closely related genes in the phylogenetic tree shared similar structure compositions. The online software MEME was used to analyze the motif distribution within the *CcSAD* genes. A total of 6 motifs were identified, and the results showed that each CcSADs contained 4 or 6 motifs (**Figure 2C**). Some motifs were common to all members, such as motif3, motif4, motif5, and motif6. The motif2 and motif3 were residues conserved that were related closely with the homodimer interface of acyl-ACP desaturase. The motif5 was also residues conserved related closely with fatty acid desaturase_2.

### Promoter cis-element and transcription factor analysis

**Figure 3:**
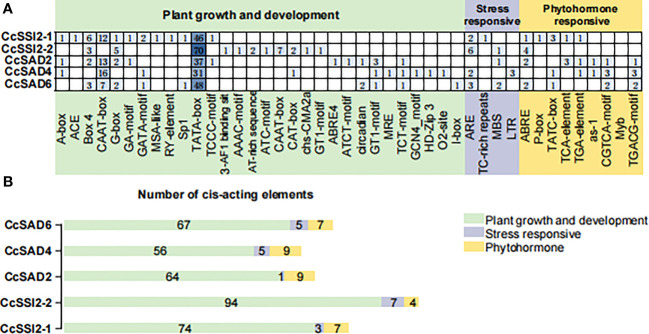
Analysis of cis-acting elements in the promoter regions of *CcSADs.* **(A)** The distribution of cis-acting elements in the promoter region of CcSAD gene family members. **(B)** The number of cis-acting elements in the promoter regions of *CcSAD* gene family members. Note, the cis-acting regulatory elements were identified in the upstream 2,000-bp sequences of the genes using the PlantCARE website. These cis-acting elements are implicated in hormone signaling, stress responses, and plant development, which are highlighted in different colors.

Analyzing promoter cis-elements offers valuable information on tissue-specific gene expression and stress response modes. More than 40 cis-elements were detected which were related to environmental stress, hormone-responsive, light-responsive, development, site binding, and other functions (**Figures 3A**, **B**), suggesting complex networks in regulation of *CcSADs*. There were four elements related to responses to environmental stress in *CcSADs* promoters: ARE (ABA-responsive element), TC-rich repeat, MBS (Myb binding site), and LTR (Low temperature responsive). Hormone-responsive elements were widely distributed in the upstream regulatory sequence of the *CcSAD* gene, including abscisic acid-responsive elements (e.g., ABRE), auxin-responsive elements (e.g., TGA-element), gibberellin -responsive elements (e.g., P-box, TATC-box), MeJA-responsive elements (e.g., CGTCA-motif, TGACG-motif), and salicylic acid-responsive elements (e.g., TCA-element).

### Subcellular location of CcSADs proteins

**Figure 4:**
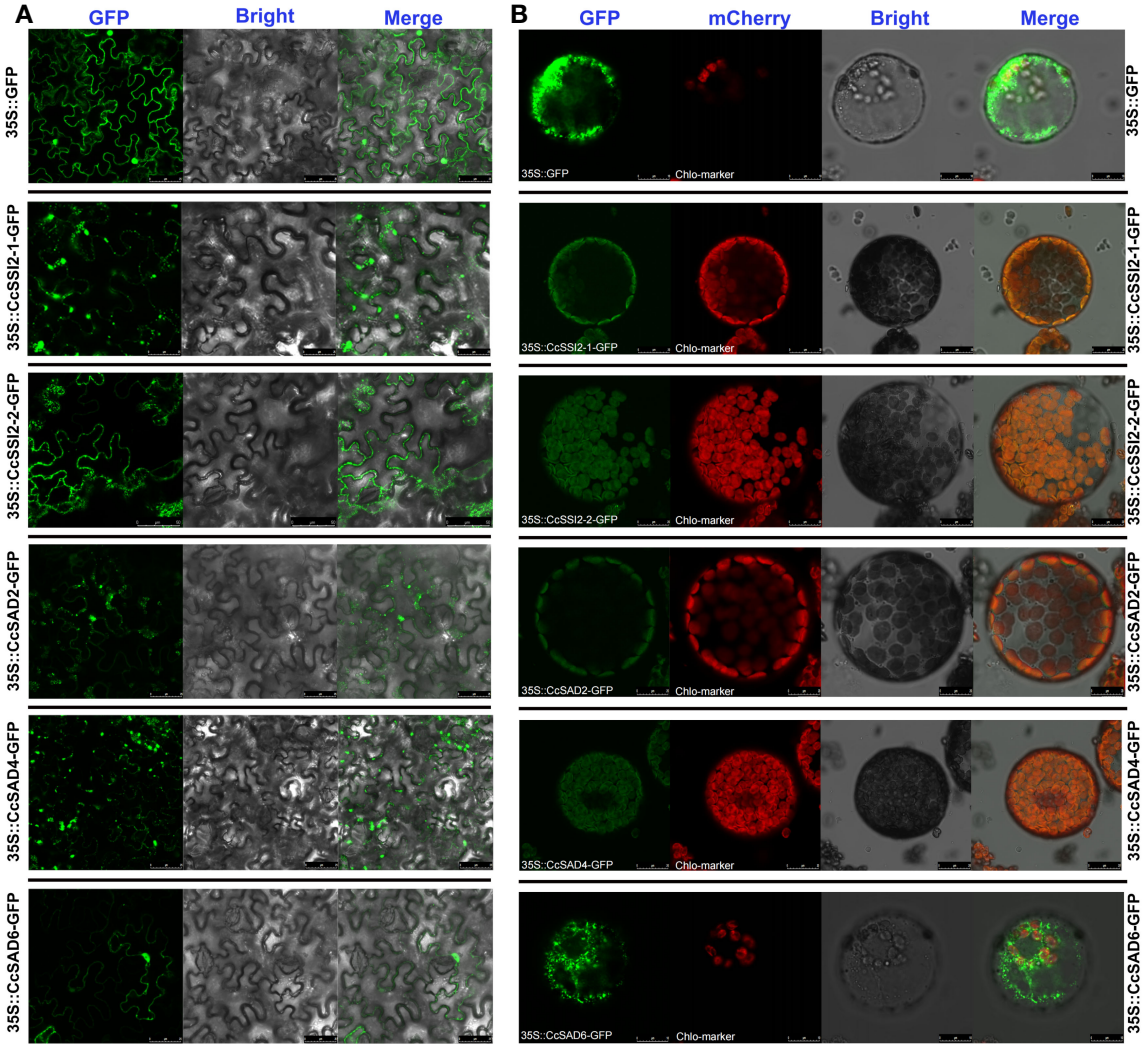
Subcellular localization of CcSAD genes in tobacco leaves and *Arabidopsis* protoplasts. **(A)** Subcellular localization of CcSAD genes in tobacco leaves where GFP stands for Green Fluorescent Protein, and it is a protein that emits green fluorescence when exposed to specific light. Merge is a term used to describe the process of combining GFP and bright field images into a single image. **(B)** Subcellular localization of CcSAD genes in *Arabidopsis* protoplasts. *Arabidopsis* protoplasts express the SAD-GFP fusion protein observed under a laser scanning confocal microscope. RUB1sp was used as chloroplast localization marker fusion with mCherry.

To verify predicted results on the subcellular localization of CcSAD proteins, full-length CDSs of all CcSAD genes were successfully cloned into the pCAMBIA1300-EGFP vector. Results showed that the uninserted GFP protein was expressed in various organelles in *N. benthamiana*. However, the CcSAD-GFP fusion proteins (CcSAD2, CcSAD4, CcSSI2-1, and CcSSI2-2) were only expressed in the chloroplast, suggesting that these CcSADs were expressed and functional in the chloroplast (**Figure 4A**). In contrast, the green fluorescence signal from CcSAD6-GFP was localized in cytoplasm which was due to the absence of chloroplast signal peptide. In this experiment, the CcSAD-GFP and RUB1sp-mCherry chloroplast marker genes were co-transformed into Arabidopsis protoplasts. The protoplasts were then scanned for green and red fluorescence, respectively. When the SAD-GFP protein was localized in chloroplasts, it overlapped with a chloroplast marker to produce a yellow fluorescence signal (**Figure 4B**). This was due to the combination of the green fluorescence from CcSAD-GFP and the red fluorescence from the chloroplast marker. The results were consistent with the transient expression observed in tobacco.

### Molecular docking

**Figure 5:**
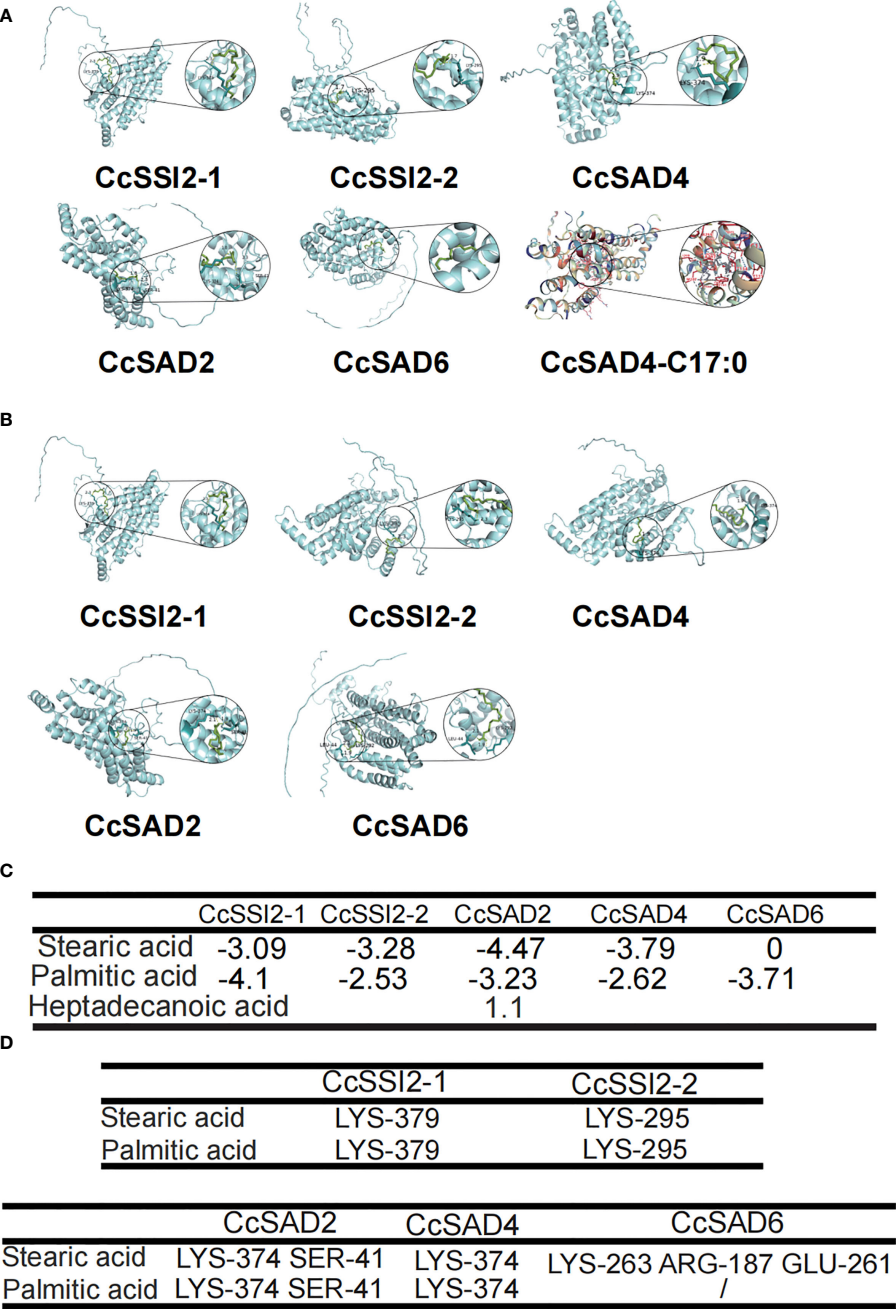
Molecular docking analysis of CcSAD proteins. **(A)** CcSAD proteins docked to stearic acid. **(B)** CcSAD proteins docked to palmitic acid. The overlay of the crystal structure of CcSAD proteins were predicted by AlphaFold2. Structure-based docking analysis was carried out using AutoDock Vina. **(C)** Energy of CcSAD protein-substrate docking. **(D)** Residues of CcSAD protein docked to the substrate.

Molecular docking is a computational technique aiming at predicting the favored orientation of a protein or a ligand to its macromolecular target (receptor) through energy matching, spatial matching, and chemical matching; when the protein and its receptor are bound together to form molecular complexes, the complex structures could be predicted (Salmaso and Moro, 2008). In short, it is the process of placing a ligand molecule into the active site of a protein macromolecule, allowing to observe the conformation of the small molecule bound to the protein and predicting the energy of action. The 3D structure of the CcSAD proteins predicted by Alphafold2 is presented in **Figure 5**. **Figures 5A, B** showed the specific position of stearic acid and palmitic acid binding with CcSAD proteins, respectively. The binding energies of the ligands docked to the target proteins are described in **Figure 5C**. The lower the binding energy indicates the more substantial the docking of the protein to the ligand. The best binding affinity was predicted to be −4.47 kcal/mol for the conformation represented in CcSAD2- stearic acid-binding. All SAD proteins had a strong binding capacity for stearic acid and palmitic acid, except for CcSAD6, which had a weak affinity for stearic acid. Heptadecanoic acid (17:0) exhibited an insufficient binding capacity to CcSAD4 as it is not present in plants. The amino acid residues presented in the **Figures 5A, B** were predicted to interact with their substrates, meaning that the residues binding with stearic acid or palmitic acid were considered to be the predicted binding pocket identified through the molecular docking studies.

### Expression patterns of *CcSADs* genes in *C. cathayensis* organs

**Figure 6:**
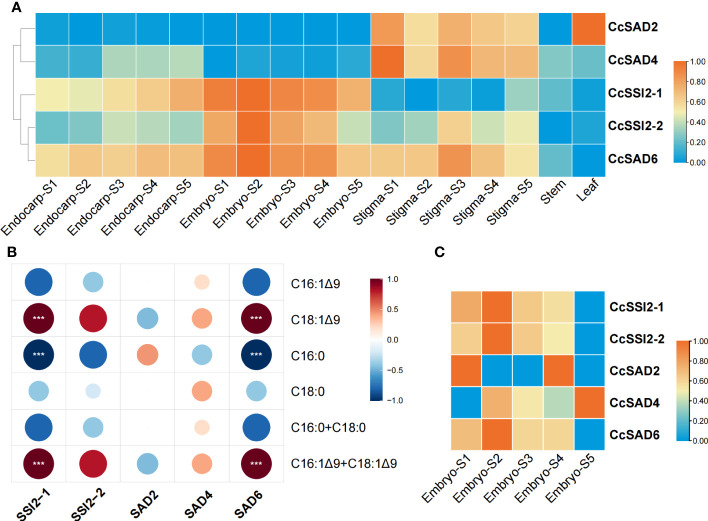
Expression profiles of *CcSAD* genes and proteins in *C*. *cathayensis.* **(A)** Different tissue expression profiles of *CcSAD* genes in *C*. *cathayensis.* The RNA-Seq data were downloaded from Huang et al. (2022a); Li et al. (2022b), and Xing et al. (2022).The expression was measured as fragments per kilo-base transcript per million mapped reads (FPKM) based on the Illumina HiSeq platform. **(B)** Correlation analysis of unsaturated fatty acid changes and SAD gene expression. **(C)** Different expression profiles of CcSAD proteins in *C*. *cathayensi.* The color bar represents the original expression value, which was log2 transformed for normalization. Red color in the scale bar indicates genes/proteins with high levels of expression and blue for genes/proteins with low levels of expression. "***", denotes p<0.05.

The expression level of each *CcSADs* gene in different tissues or organs, including endocarp, embryo, stigma as well as stems and leaves (**Figure 6A**) was analyzed. The results revealed that five *CcSAD* genes had diverse expression patterns among organs during *C. cathayensis* development. *CcSAD6, CcSAD2*, and *CcSAD4* were mainly expressed in different developmental stages of stigma, and *CcSAD2* was highly expressed in leaves. Genes overexpressed in embryo were *CcSAD6, CcSSI2-1*, and *CcSSI2-2*. Moreover, *CcSAD6* and *CcSSI2-1* were highly expressed in the endocarp. These results suggested that these *CcSAD* genes could play different roles in the development of different organs although their sequences were highly similar. Correlation analysis of unsaturated fatty acid contents during embryo development with the level of *SAD* gene expression (**Figure 6B**) showed that the accumulation of oleic acid (C18:1) was highly correlated with *CcSSI2-1* and *CcSAD6* gene expression (r > 0.9) and also correlated with *CcSSI2-2* (r > 0.8). The expression patterns of the *CcSAD* gene differed in different tissues of hickory. *CcSSI2-1*, *CcSSI2-2*, and *CcSAD6* were highly expressed in the embryo at the S2 stage, which corresponded to the rapid accumulation of hickory oil. Moreover, *WRI1*, a transcription factor involved in lipid regulation, also exhibited high expression at the S2 and S3 phases of hickory, suggesting that they may potentially regulate *SAD* genes in hickory (Huang et al., 2022a). The transcripts and proteins of CcSSI2-1, CcSSI2-2, and CcSAD6 were all expressed during embryo development, and they all were expressed at the highest level during the rapid accumulation of oil (S2) and then slowly decreased. The protein expression patterns of the *CcSADs* were also found to be consistent with RNA-Seq results (**Figure 6C**). Thus, *CcSAD6, CcSSI2-1*, and *CcSSI2-2* were mainly expressed in embryos with the highest expression at the early developmental stage, indicating that these genes played important roles in regulation of oleic acid biosynthesis in embryos.

### Analysis of the fatty acid composition of different organs

**Figure 7:**
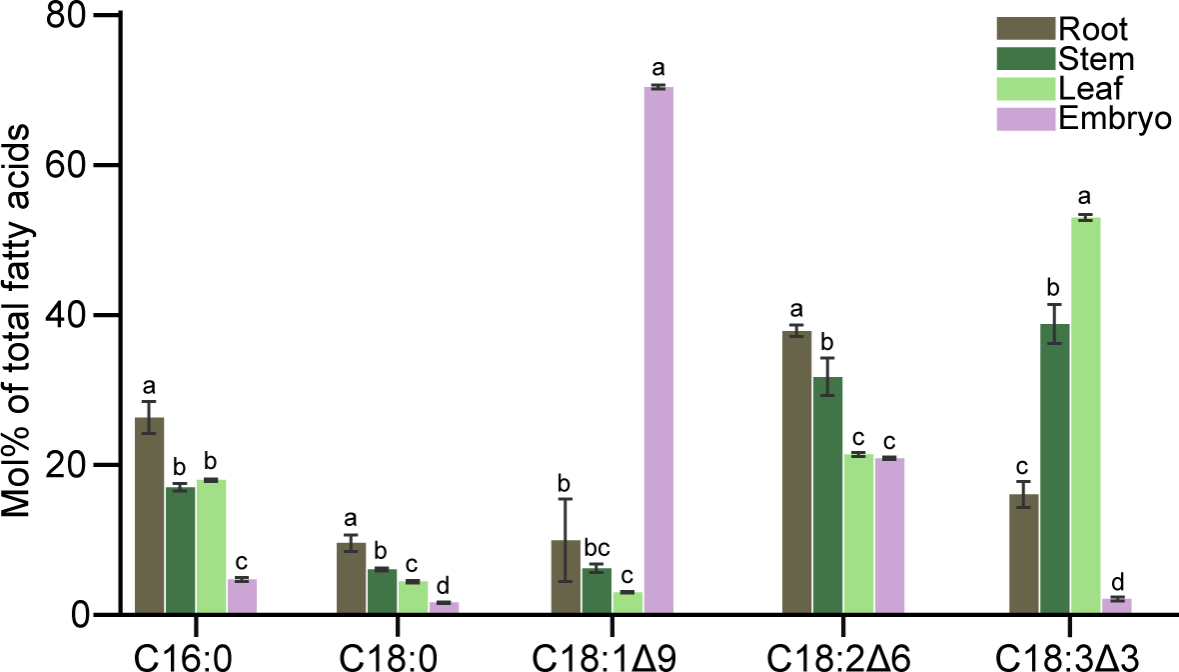
Fatty acid composition in roots, stem, leaves, and embryos of *C. cathayensis.* Values represent the means plus standard deviations of three biological replicates. Student’s *t*-test was used to analyze the difference between samples in the percentage of each fatty acid. ^a^ denotes p < 0.05.

Fatty acid compositions in roots, stems, leaves, and embryos were analyzed by GC-FID (**Figure 7**). Unsaturated fatty acids were higher than saturated fatty acids regardless of organs. In roots, saturated fatty acids (C16:0 and C18:0) were approximately 30% of all fatty acids; whereas C18:2 as the major unsaturated fatty acid accounted for more than 30% of total fatty acids. In stem tissue, unsaturated fatty acids were also the major component of the total fatty acids, with both C18:2 and C18:3 accounting for more than 30% of the total fatty acids. The fatty acid composition in leaves was similar to that of the stem tissue. It was noteworthy that the C18:1 content in embryo was almost 70% of the total fatty acids, and the C18:2 content was more than 20%.

### Functional validation of SAD proteins

**Figure 8:**
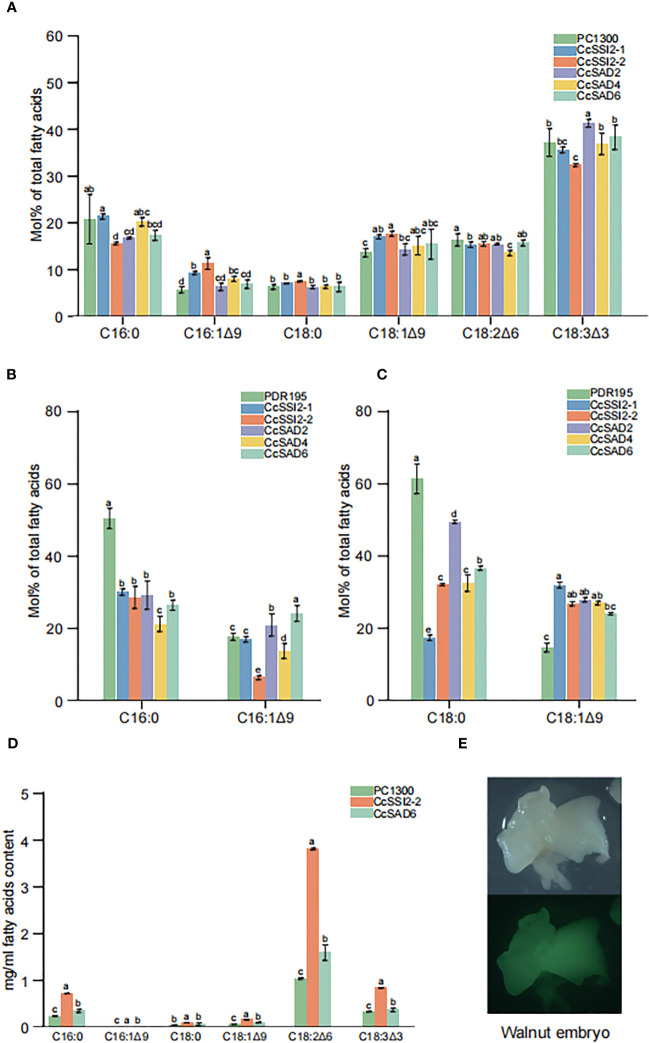
Functional validation of the *CcSAD* genes. **(A)** Fatty acid (FA) compositions in tobacco leaves resulting from the overexpression of 35S::CcSADs. **(B)** FA compositions due to the overexpression of 35S::CcSADs in yeast BY4839 with C16:0 as the substrate. **(C)** FA compositions resulting from the overexpression of 35S::CcSADs in yeast BY4839 with C18:0 as the substrate. **(D)** FA compositions in walnut embryos due to the overexpression of 35S::CcSADs. **(E)** Expression of GFP protein in walnut embryos. Values represent the means plus standard deviations of three biological replicates where Student’s *t*-test was used to analyze the differences between samples in the percentage of each fatty acid. ^a^ denotes p < 0.05.

To investigate the function of SAD proteins, their expressions in different cells and their effects on fatty acid compositions were examined. The transient expression in tobacco leaves was confirmed with laser confocal microscopy. Results showed that the total fatty acid content in tobacco leaves was low with C18:3 as the primary fatty acid accounting for more than 30% of the total fatty acids in leaves. Transient expression of *CcSSI2-1* and *CcSSI2-2* resulted in significant increase in C16:1 and C18:1. The expression of *CcSAD2, CcSAD4*, and *CcSAD6* also led to the high unsaturated fatty acid content in tobacco leaves (**Figure 8A**). *CcSAD* genes were also expressed in yeast BY4389, during which C16:0 or C18:0 was added to the medium as a substrate for detecting changes in fatty acids after co-culture. **Figure 8C** shows that the expression of the hickory *SAD* gene in BY4389 reduced the proportion of C18:0 and increased the proportion of C18:1 from 15% to 30%, and *CcSAD6* also increased the content of C16:1 (**Figure 8B**). It is known that genetic transformation of woody plants has been a challenging issue. The polyphenol content in the embryo of hickory is extremely high, which makes it impossible to establish an effective tissue culture system. In this study, we transformed *SAD* genes into walnut embryos. Walnuts are genetically close to hickory, have high oil content in the embryo, and are rich in unsaturated fatty acids. We analyzed transient expression of *SAD* genes in walnut embryos coupled with fluorescence detection (**Figures 8D**, **E**). Transient expression of *CcSSI2-1* and *CcSAD6* in walnut embryos revealed that the major fatty acids in walnut embryos were polyunsaturated fatty acids (C18:2 and C18:3) in which *CcSSI2-1* increased the content of C18:2 while *CcSAD6* promoted the synthesis of C18:3. Although the level of C18:0 in walnut embryos was low, both *CcSSI2-1* and *CcSAD6* were able to promote the biosynthesis of C18:1.

## Discussion

The *SAD* gene family has been characterized in several plant species, including seven genes in *A. thaliana*, four genes in *Cynara cardunculus* (Cappetta et al., 2022), 11 genes in maize (Han et al., 2017), 19 genes in *Gossypium hirsutum* (Mo et al., 2021), and eight gene in *Theobroma cacao* (Zhang et al., 2015). They not only regulate fatty acid biosynthesis (Knutzon et al., 1992; Perez-Vich et al., 2002) but also mediate plant growth and responses to abiotic and biotic stresses (Cheesbrough, 1990; Schluter et al., 2011; Klinkenberg et al., 2014; Peng et al., 2018). However, there has been no comprehensive analysis of *SAD* genes in *C. cathayensis* and their effects on OA biosynthesis.

The present study identified five *SAD* genes in Chinese hickory through the genome-wide search. Subsequently, gene structures, expression profiles, phylogenetic relationships, and conserved motifs were investigated. According to the evolutionary relationship inferred by phylogenetic analysis, the five members were divided into two clades, which was consistent with those in *Arabidopsis* (Kachroo et al., 2007), olive (Parvini et al., 2016), and potato (Li et al., 2015a). A comprehensive analysis of the gene structure revealed that the numbers of introns were either one or two among *CcSADs*, similar to the exon/intron organization of *SAD* genes reported in cacao (Zhang et al., 2015). Like other plants, CcSADs are hydrophilic proteins with a grand hydropathicity from -0.22 to -0.459 (Kachroo et al., 2007; Zhao et al., 2015; Li et al., 2022a). The conserved domain plays an important role in protein structure, transcriptional activity, protein subcellular localization, biological function, and protein evolution (Vogel et al., 2004). The hydrophobicity plot analysis using ProtScale indicated that CcSADs were water-soluble proteins without transmembrane domains, which concurred with the property as soluble desaturases. Protein domain analysis showed that CcSADs, except for CcSAD6, contained a conserved Acyl_ACP_Desat domain, a FA desaturase domain as well as a ferritin superfamily member that had two iron atoms at the N-terminus. Two highly conserved E/DEXXH motifs were found in the diiron-oxo protein, which is considered to be critical to the activity of soluble plant desaturases (Lindqvist et al., 1996). Each stearoyl-ACP desaturase is known to require two iron atoms for the formation of Fe–O–Fe complex as the center for catalytic reactions (Shanklin and Somerville, 1991; Shanklin et al., 1994). Crystal structure studies confirm that the two iron atoms interact with side chains of E196 H232 and E105 H146 (Lindqvist et al., 1996). These conserved amino acids were also found in CcSAD2, CcSAD4, CcSSI2-1, and CcSSI2-2. CcSSI2-1 and CcSSI2-2 were homologous to *A. thaliana* SSI2 (SUPPRESSOR OF SA-INSENSITIVITY2; At2g43710) (Song et al., 2013). This study also simulated the interaction between CcSAD proteins and fatty acid substrates through the prediction of the crystal structure of CcSADs and combination of the Autodock tool with molecular docking. The principle of the molecular docking is based on the fact that these residues are involved in water translocation and the selection of other substrate molecules, such as glycerol or FAs. A triple mutant of stearoyl-acyl carrier protein desaturase (T117R/G188L/D280K) from castor bean (*Ricinus communis*) was also used to investigate the catalytic activities of each amino acid (Whittle et al., 2020).

The CcSAD2, CcSAD4, CcSSI2-1, and CcSSI2-2 were predicted to be chloroplast transit peptides (cTPs) that started at the N-terminus. These results were confirmed by transient expression of CcSADs-GFP in tobacco leaves and *Arabidopsis* protoplasts, suggesting that they may play important roles in the desaturation of fatty acids in chloroplasts. Other experimental evidence also indicates that SADs are indeed localized in chloroplasts (Li et al., 2022a; Yang et al., 2022). The lack of chloroplast signaling at the N-terminal end of the CcSAD6 protein also renders that it is unavailable for expression in chloroplasts. In addition, dark treatment decreased transcript abundance, along with a decrease in the UFA content of chloroplast lipids as well as olive oil quality, which may also indicate SDAs’ location in chloroplast (Hernandez et al., 2019).

Promoter DNA sequences determine the variability of gene expression in which cis regulatory elements play critical role (Einarsson et al., 2022). This study showed that promoter regions of *CsSADs* have over 40 cis-acting elements, including CAT-box, RY-element, MBS, ABRE, CGTCA-motif, and TGACG-motif as well as TCA element and TATC box. Such a large number and diversity of cis-acting elements may suggest that *CsSADs* are engaged in plant growth and development as well as responses to abiotic and biotic stresses. The CAT-box in the promoter regions of *CcSSI2-2* and *SAD4* is implicated in meristem expression. RY-element in the upstream region of *CcSSI2-1* is involved in seed-specific regulation. MBS is the Myb binding site that is known to regulate tissue-specific expression of genes, and the MYB target sequence may be also responsible for the organ-specific expression of *SADs* in *C. cathayensis*. For example, *CcSAD6, CcSAD2*, and *CcSAD4* are primarily expressed in stigma, *CcSAD2* is expressed in leaves; *CcSAD6, CcSSI2-1*, and *CcSSI2-2* in embryos. TGACG and CGTCA motifs are responsible for jasmonte, and ABRE perceives ABA signal; these cis-regulatory elements in *SADs* could recognize abiotic signals, regulating plant tolerance to drought or extreme temperatures. Thus, the availability of over 40 cis-acting elements in the promoter region of different *CcSADs* could enable *C. cathayensis* to better respond to various chemical and environmental signals. Similar findings were reported in olive plants where *SAD* genes differentially responded to wounding, resulting in the modifications of UFA compositions.

An important finding of this study is the documentation of the role of individual *CcSADs* in regulation of OA accumulation in *C. cathayensis* kernel. Previous studies have shown that high OA content in some crops is related to the accumulation of SAD transcripts (Li et al., 2015b; Huang et al., 2017; Lin et al., 2018; Liu et al., 2019b; Yang et al., 2022). Multi-omics studies with hickory also suggested the possible importance of *SAD* genes in the accumulation of OA during embryo development (Huang et al., 2016; Huang et al., 2022a). These studies, however, did not identify gene numbers of SAP and their specific role in the production of OA. In this study, the combined transcriptomic and proteomic analyses showed that *CcSSI2-1, CcSSI2-2*, and *CcSAD6* were expressed during embryo development, and both transcript and protein expression indicated that OA accumulation progressed with the development of kernel development and then decreased before kernel maturation. It was likely that the first double bond was introduced to saturated fatty acids by SAD enzymes, resulting in the modification of the composition of stearic and oleic acid. In olive plants, *OeSAD2* was found to be the major gene contributing to the elevated levels of oleic acid content (Parvini et al., 2016). In the present study, *CcSSI2-1* and *CcSSI2-2* were found to be highly expressed in embryo development. A comprehensive transcriptomic analysis of *Camellia oleifera* showed that OA accumulation in seeds was associated with higher levels of stearoyl-ACP desaturases (SADs) and lower fatty acid desaturase 2 (FAD2) activities (Lin et al., 2018). Similarly, the high proportion of OA in total fatty acids was reported to be determined by high activity of stearoyl-ACP desaturase (SAD) in the chloroplast and/or rather lower FA desaturase (FADs) activity in the ER (Bates et al., 2013). Our studies with CcSADs-green fluorescent protein (GFP) fusion constructs also indicated that *CcSADs* were localized in chloroplasts as well. This study further endorses that CcSAD can use both C16:0 and C18:0 as substrates, but different members of the CcSAD genes do not have similar substrate recognition activity. The catalytic function of these *CcSADs* was analyzed by heterologous expression in *S. cerevisiae*, *N. benthamiana*, and walnut, which demonstrated that all CcSADs possess fatty acid desaturase activity to catalyze oleic acid biosynthesis, and some members of CcSADs also have strong substrate specificity for 16:0-ACP to synthesize palmitoleic acid (C16:1, PA).

Unsaturated fatty acids like OA are much preferred oil for human consumption as they can reduce blood cholesterol levels, improve blood cycle, and reduce the risk of heart diseases (Lunn and Theobald, 2006). Nuts of *C. cathayensis* represent a valuable source of unsaturated fatty acids as its kernel is composed of 90% monounsaturated and polyunsaturated fatty acids of which OA accounting for more than 70%. The biosynthesis of OA in plants has been extensively studied for increasing the feasibility of efficient genetic engineering of fatty acids composition in a wide range of plants, and *SADs* are considered key candidate genes for manipulation. Overexpression of *XsSAD* in the *Arabidopsis ssi2* mutant effectively increased the level of 18:1 (Zhao et al., 2015). Under seed-specific overexpression of *ZmSAD1* in Arabidopsis, the stearic acid (C16:0) content and the ratio of saturated to unsaturated fatty acids in the seeds were significantly reduced (Du et al., 2016).

Heterologous expression of *PoSAD*, a gene from *Paeonia ostii* significantly decreased SA and increased OA content in *S. cerevisiae* and *A. thaliana* (Li et al., 2020). However, to effectively engineer *SAD* genes for high OA production, *SADs* from high OA content plants, such as *C. cathayensis* could be an ideal choice. In this study, we documented that CcSSI2-1, CcSSI2-2, and CcSAD6 were highly expressed in kernels. These genes could be appropriate candidate genes for improving OA contents in *C. cathayensis* and other nut crops through either genetic transformation or CRESPR/Cas9 technologies.

## Conclusion

The present study identified the *SAD* gene family in *C. cathayensis.* Five *CcSAD* isoforms in the hickory genome shared a high degree of amino acid sequence conservation but also had specific differences in key determinant amino acid residues. Over 40 cis-acting elements were situated in the promoter regions, which allow to mediate distinct tissue-specific expression patterns of these genes. RNA-Seq analysis showed that *CcSSI2-1*, *CcSSI2-2*, and *CcSAD6* were highly expressed in kernels. The expression pattern of the CcSADs during kernel development was also significantly correlated with fatty acid and oil accumulation. In addition, five full-length cDNAs encoding SAD were isolated from the developing kernel of *C. cathayensis*. CcSADs-green fluorescent protein (GFP) fusion constructs were expressed in tobacco epidermal cells and *Arabidopsis* protoplasts, which provide clear evidence about their chloroplast localization. The catalytic function of these *CcSADs* was further analyzed by heterologous expression in *S. cerevisiae*, *N. benthamiana*, and walnut. Results indicate that all CcSADs possess fatty acid desaturase activity to catalyze OA biosynthesis. Some members of CcSADs also have strong substrate specificity for 16:0-ACP to biosynthesize palmitoleic acid (C16:1, PA). As far as is known, this is the first documentation of SAD gene family in *C. cathayensis*. The highly expressed *CcSSI2-1*, *CcSSI2-2*, and *CcSAD6* in embryos may suggest that they are probably the target genes for improving oil composition in crops through genetic manipulation. Oils with a high level of oleic acid have a better resistance to thermal and oxidation degradation. Long-term consumption of high oleic acid oil can lower the level of low-density lipoprotein (LDL) cholesterol while preserving high-density lipoprotein (HDL) cholesterol in the human body, thereby reducing risks of cardiovascular diseases and stroke. Therefore, exploring the catalytic activity of Δ9 fatty acid desaturase gene family should improve our understanding of the biosynthetic mechanism of oleic acid and help develop high-oleic acid crops through genetic modification.

## Data availability statement

The original contributions presented in the study are included in the article/**Supplementary Material**. Further inquiries can be directed to the corresponding authors.

## Author contributions

KW designed the experiments. SL and XS performed the experiments. JH and YL analyzed the data. QH, CH and HY mapped all the figures. SL wrote the manuscript. JC edited and refined the manuscript. All authors contributed to the article and approved the submitted version.
