# Supplementary material for: Analysis of Delta(9) fatty acid desaturase gene family and their role in oleic acid accumulation in Carya cathayensis kernel
Source: Front Plant Sci. 2023 Sep 12;14:1193063. doi: 10.3389/fpls.2023.1193063 (PMC10523321; doi:10.3389/fpls.2023.1193063)
Supplement: Supplementary file 1 [file Table_1.docx]

Primers used for construction of yeast expression vector

195-SSI2-1-F:cctcgagcggccgcgATGGCTCTCAAACTCTGTTTTT

195-SSI2-1-R:agaagtccaaagctgGAGCTTCACATTTCTATCAAAA

195-SSI2-2-F:cctcgagcggccgcgATGGCTCTCAAACTCGGTATTTTC

195-SSI2-2-R:agaagtccaaagctgGAGCTTCACTTCTCTATCAAAAATCCA

195-SAD2-F:cctcgagcggccgcgATGCAGATGCAGGCCTTACACT

195-SAD2-R:agaagtccaaagctgTAATTTGACCTCCCTATTGAAA

195-SAD4-F:cctcgagcggccgcgATGGATTTAAAGCTCATGAACAGCT

195-SAD4-R:agaagtccaaagctgCATGCCAGAAATCCAACTGAAGG

195-SAD6-F: cctcgagcggccgcgATGCTTAATACCCTGGATGGAGTT

195-SAD6-R: agaagtccaaagctgGCAAGTAGCAACAGGAGGAACAT

Primers used for construction of plant GFP expression vector

GFP-SAD2-F:agctcggtacccgggATGCAGATGCAGGCCTTACACT

GFP-SAD2-R:atgtcgactctagagTAATTTGACCTCCCTATTGAAA

GFP-SSI2-1-F:agctcggtacccgggATGGCTCTCAAACTCTGTTTTT

GFP-SSI2-1-R:atgtcgactctagagGAGCTTCACATTTCTATCAAAA

GFP-SSI2-2-F:agctcggtacccgggATGGCTCTCAAACTCGGTATTTTC

GFP-SSI2-2-R:atgtcgactctagagGAGCTTCACTTCTCTATCAAAAATCCA

GFP-SAD4-F:agctcggtacccgggATGGATTTAAAGCTCATGAACAGCT

GFP-SAD4-R:atgtcgactctagagCATGCCAGAAATCCAACTGAAGG

GFP-SAD6-F:cagctcggtacccgggATGCTTAATACCCTGGATGGAGTT

GFP-SAD6-R:atgtcgactctagagGCAAGTAGCAACAGGAGGAACAT
